# Supplementary material for: On the Effect of Standard Deviation of Cationic Radii on the Transition Temperature in Fluorite-Structured Entropy-Stabilized Oxides (F-ESO)
Source: Materials (Basel). 2023 Mar 10;16(6):2219. doi: 10.3390/ma16062219 (PMC10056515; doi:10.3390/ma16062219)
Supplement: Supplementary file 1 [file materials-16-02219-s001.zip › materials-2227420-supplementary.pdf]

materials

*Supplementary Material*

# On the effect of standard deviation of cationic radii on the transition temperature in Fluorite-structured Entropy-Stabilized Oxides (F-ESO)

**Luca Spiridigliozzi [1,*], Mauro Bortolotti [2] and Gianfranco Dell'Agli [1,3]**

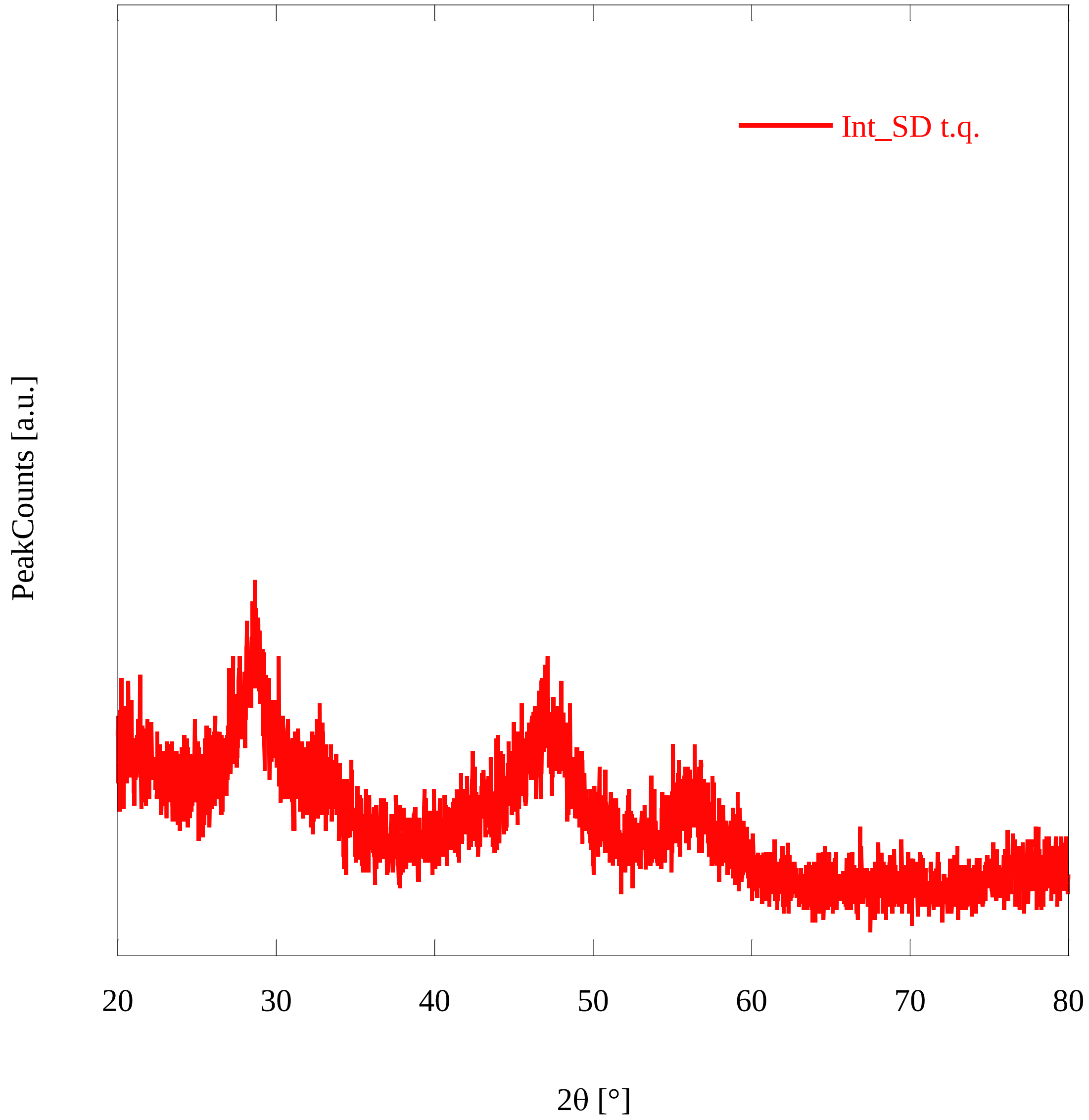


**Figure S1.** Diffraction pattern of as-prepared sample Int_SD (t.q.).

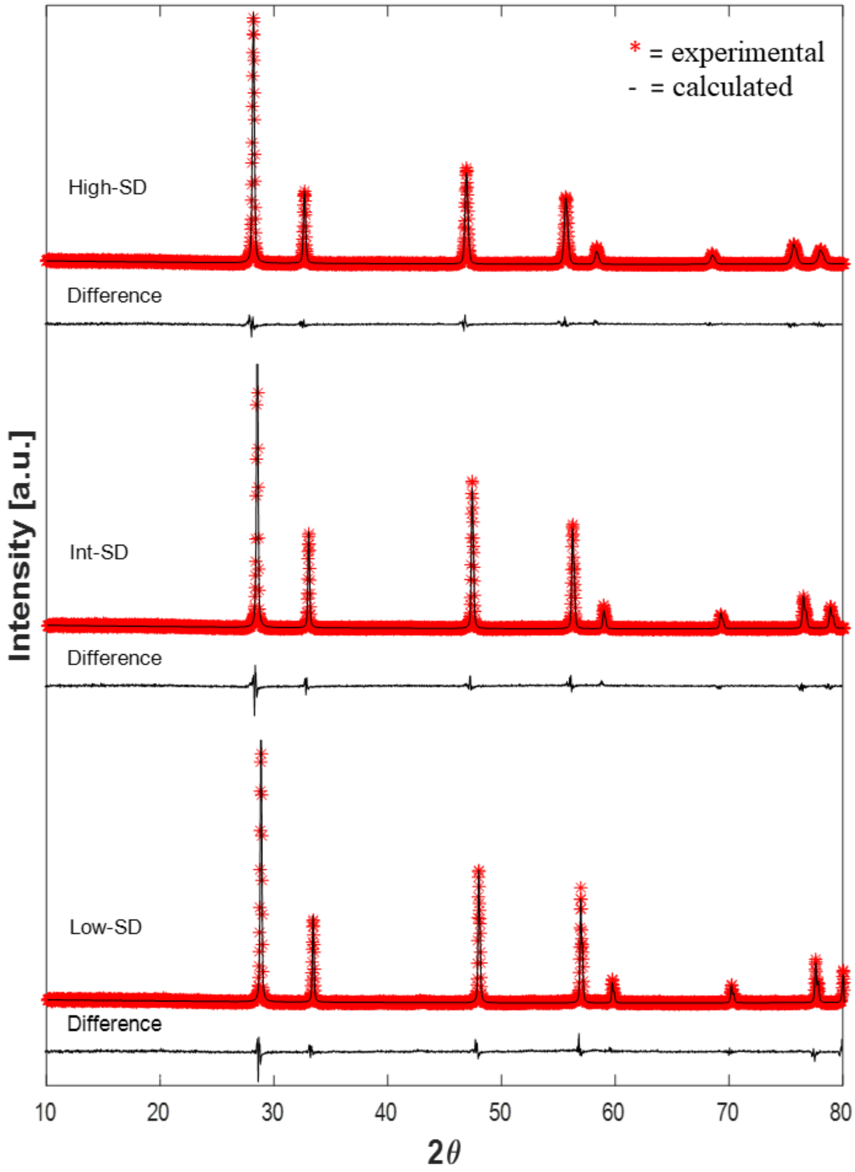


**Figure S2.** Rietveld refinements of High_SD, Int_SD, and Low_SD
